# A consecutive case series experience with [^18^ F] florbetapir PET imaging in an urban dementia center: impact on quality of life, decision making, and disposition

**DOI:** 10.1186/1750-1326-9-10

**Published:** 2014-02-03

**Authors:** Effie M Mitsis, Heidi A Bender, Lale Kostakoglu, Josef Machac, Jane Martin, Jennifer L Woehr, Margaret C Sewell, Amy Aloysi, Martin A Goldstein, Clara Li, Mary Sano, Sam Gandy

**Affiliations:** 1 Department of Psychiatry, Icahn School of Medicine at Mount Sinai, One Gustave L. Levy Place, Box 1230, New York, NY 10029, USA; 2 Department of Neurology, New York, NY 10029, USA; 3 Department of Nuclear Medicine, New York, NY 10029, USA; 4 Icahn School of Medicine at Mount Sinai, Alzheimer’s Disease Research Center, One Gustave L. Levy Place, Box 1230, New York, NY 10029, USA; 5 James J. Peters Veterans Affairs Medical Center, Bronx, NY 10468, USA

**Keywords:** Amyvid™, Florbetapir, PET, Clinical series, Alzheimer’s disease, Neuroimaging

## Abstract

**Background:**

Identification and quantification of fibrillar amyloid in brain using positron emission tomography (PET) imaging and Amyvid™ ([^18^ F] Amyvid, [^18^ F] florbetapir, ^18^ F-AV-45) was recently approved by the US Food and Drug Administration as a clinical tool to estimate brain amyloid burden in patients being evaluated for cognitive impairment or dementia. Imaging with [^18^ F] florbetapir offers *in vivo* confirmation of the presence of cerebral amyloidosis and may increase the accuracy of the diagnosis and likely cause of cognitive impairment (CI) or dementia. Most importantly, amyloid imaging may improve certainty of etiology in situations where the differential diagnosis cannot be resolved on the basis of standard clinical and laboratory criteria.

**Results:**

A consecutive case series of 30 patients (age 50-89; 16 M/14 F) were clinically evaluated at a cognitive evaluation center of urban dementia center and referred for [^18^ F] florbetapir PET imaging as part of a comprehensive dementia workup. Evaluation included neurological examination and neuropsychological assessment by dementia experts. [^18^ F] florbetapir PET scans were read by trained nuclear medicine physicians using the qualitative binary approach. Scans were rated as either positive or negative for the presence of cerebral amyloidosis. In addition to a comprehensive dementia evaluation, post [^18^ F] florbetapir PET imaging results caused diagnoses to be changed in 10 patients and clarified in 9 patients. Four patients presenting with SCI were negative for amyloidosis. These results show that [^18^ F] florbetapir PET imaging added diagnostic clarification and discrimination in over half of the patients evaluated.

**Conclusions:**

Amyloid imaging provided novel and essential data that: (1) caused diagnosis to be revised; and/or (2) prevented the initiation of incorrect or suboptimal treatment; and/or (3) avoided inappropriate referral to an anti-amyloid clinical trial.

## Introduction

Diagnosis and treatment of Alzheimer’s disease (AD) has been hindered by the lack of affirmative, non-invasive *in vivo* measures to identify the hallmark neuropathology of the disease. To date, an accurate and definitive diagnosis of AD can only be determined at postmortem examination. The reliance on pathological reports places living patients seeking accurate diagnosis, and ultimately appropriate treatment, at risk for misidentification of disease due to overlap in clinical presentation, especially in early stages of disease. Further, the absence of an affirmative diagnostic test can lead to uncertainty and psychological distress among patients and caregivers, due to equivocal findings that are ultimately costly and frustrating. Binary reading of retention of Amyvid™ (also known as [^18^ F] florbetapir; ^18^ F-AV-45; [^18^ F] Amyvid) has been approved recently by the US Food and Drug Administration (FDA) as a clinical tool for physicians to estimate cerebral fibrillar amyloid burden in patients being evaluated for cognitive impairment or dementia. Imaging with [^18^ F] florbetapir could potentially: (1) increase confidence and accuracy of the clinical diagnosis of AD; (2) rule out the presence of amyloid thereby suggesting an alternative cause of CI; and (3) clarify confusion due to the overlap between symptoms of AD and those of other neurodegenerative diseases.

Many patients with AD go undiagnosed within primary care settings [1]. Fewer than half of patients with dementia have a documented diagnosis in their primary care medical records, especially in milder or earlier stages of disease [2]. The use of biomarkers that identify amyloid pathology could lead to more confident diagnosis and symptom management by primary care physicians as well as specialists. We present here the impact of [^18^ F] florbetapir imaging on clinical practice related to the diagnosis and treatment of patients being evaluated for cognitive decline.

Research studies have shown that [^18^ F] florbetapir has high affinity and specificity to fibrillar assemblies of the amyloid-β peptide [3]. The ligand enters the brain quickly once injected, demonstrates separation between individuals with and without amyloid within 30 minutes, and plateaus within 50 minutes [3]. A 10-minute scan at 50-60 minutes post-injection is considered optimal. [^18^ F] florbetapir has a 110-minute half-life, allowing a substantial time frame for delivery to imaging sites. The relatively brief 10-minute scan time makes [^18^ F] florbetapir imaging ideally suited in an older and potentially frail cohort.

The use of [^18^ F] florbetapir PET imaging has been validated against neuropathology in late stage disease to ensure that the imaging signal corresponds to the underlying amyloid pathology [4,5]. In two recent prospective studies evaluating whether [^18^ F] florbetapir PET imaging performed during life is predictive of presence of cerebral amyloidosis using immunohistochemistry and silver stain at autopsy [4,6], visual binary reads (positive vs. negative for amyloid) of the imaging and postmortem results for the presence of amyloidosis were in agreement in 96% of cases in a cohort of late stage dementia patients who died within one year or less [4]. In a follow-up study in a larger sample and in individuals who reached autopsy within 24 months of [^18^ F] florbetapir PET imaging, Clark *et al.*[6], again using a binary visual read approach by trained nuclear medicine physicians, replicated their initial findings that sensitivity and specificity were 92% and 100%, respectively, in a sub-sample who had autopsy within 24 months and were 96% and 100%, respectively in a sub-sample who had autopsy within 12 months [6]. In addition to validation of the binary visual read method (which is now the standard approved for clinical use in the USA), semi-quantitative analysis with [^18^ F] florbetapir PET imaging in six regions of interest was closely correlated with postmortem amyloid burden in the patients who had autopsy within 12 and 24 months (both p < 0.0001) [6].

However, recent evaluation of the clinicoradiological correlation of amyloid imaging in early stage AD reveals that as many as 30% of patients with clinically probable AD referred for clinical trials have negative amyloid imaging despite the presence of both clinical symptoms and radiological evidence of neurodegeneration by hippocampal volumetry and PET imaging with [^18^ F] fluorodeoxyglucose [7]. This has led to the proposal that neuroimaging criteria be employed for the designation of some patients as having “amyloid-first” AD and others as having “neurodegeneration-first”AD [7].

Mount Sinai Hospital was the first site in New York State approved to conduct clinical [^18^ F] florbetapir PET imaging by expert dementia clinicians and neuroimaging specialists. Since FDA approval for clinical use in April 2012, we have conducted [^18^ F] florbetapir imaging in 30 patients using a binary read approach (positive or negative for brain amyloid) by nuclear medicine physicians trained to interpret [^18^ F] florbetapir PET scans. To our knowledge, this is the first consecutive case series demonstrating the utility of [^18^ F] florbetapir imaging in clinical practice in patients seeking evaluation at a large, urban dementia center. Unlike research studies designed either to determine validity of the technology or conducted in highly selected cohorts in which participants are rigidly screened to meet distinct study inclusion criteria (i.e., AD or MCI or healthy, age-matched controls) and exclude certain types of co-morbid disorders [4,6-12], our clinical sample presented herein are patients typically seen in our tertiary care clinical practice for dementia evaluation, and therefore the full range of illnesses/co-morbidities and complex, atypical presentations seen in a typical aging cohort is represented.

## Results and discussion

As part of the comprehensive dementia evaluation, patients received the following diagnoses: probable AD (n = 12), AD with cerebral amyloid angiopathy (CAA; n = 1), mild cognitive impairment (MCI), amnestic type (n = 1), static memory impairment (n = 1), frontotemporal lobar degeneration (FTLD), behavioral variant (n = 1), FTLD, primary progressive aphasia type (n = 1), frontotemporal dementia, behavioral variant (n = 2), frontotemporal dementia, primary progressive aphasia (n = 1), subjective cognitive impairment (SCI) (n = 4), MCI or pseudodementia due to depression or Bipolar I disorder (n = 2), cognitive impairment with history of substance abuse (n = 1), vascular dementia (n = 1), delayed post-traumatic cognitive impairment (n = 1), and Parkinson’s disease with depression (n = 1). As seen in Additional file 1: Table S1, amyloid imaging results caused diagnoses to be changed in 10 patients and clarified in 9 patients. All patients presenting with SCI were negative for amyloidosis.

This is the first consecutive case series report demonstrating the value of using [^18^ F] florbetapir PET imaging for affirmative diagnostic confirmation and/or discrimination in a clinical sample of patients seen for evaluation at an urban, dementia center. We have shown that [^18^ F] florbetapir PET imaging added diagnostic clarification in over half of the patients evaluated. In patients with AD or MCI, amnestic type, [^18^ F] florbetapir imaging was positive and consistent with clinical diagnosis, supporting the use of [^18^ F] florbetapir PET imaging for diagnostic certainty. Indeed, the term “prodromal AD” has been suggested to replace the older term MCI when evidence for cerebral amyloidosis is present [13]. [^18^ F] florbetapir imaging may also help clarify diagnosis in patients who present with clinical and cognitive profiles indistinguishable from those with AD but yet, on amyloid imaging, lack readily detectable amyloidosis.

In contrast, patients in whom the etiology was unclear and who presented with Parkinson’s disease, frontotemporal dementia of the primary progressive aphasic or behavioral variant types, post-traumatic cognitive impairment (Mitsis *et al*., in preparation), depressive pseudodementia and/or bipolar disorder I, and with SCI were negative, indicating the value of [^18^ F] florbetapir PET imaging in a tertiary, urban clinical population that included patients with varied histories, backgrounds and comorbid illnesses.

Overall, our findings support the clinical utility of [^18^ F] florbetapir PET imaging as an additional biomarker tool in the evaluation of patients with ÇI due to a variety of causes. Three unusual cases were encountered:

•Patient 1 had been given various diagnoses prior to our evaluation (FTD vs. AD). FTD is a clinical syndrome that may include primary progressive aphasia (PPA) or behavioral variant (bv). AD pathology in the right distribution can mimic PPA or FTD, bv. [^18^ F] florbetapir scan was positive, adding diagnostic clarification. Our post-scan diagnosis was clinical syndrome of FTD, behavioral variant due to Alzheimer's pathology (Additional file 1: Table S1).

•Patient 20 had experienced one significant blow to the occipital region during a sports-related activity and had an otherwise negative scan. The [^18^ F] florbetapir scan was focally positive at the point of impact (Mitsis *et al.*, in preparation).

•Patient 16 had experienced multiple concussions. Based upon this patient’s cognitive profile, three experienced dementia clinicians (SG, MS, EMM) supported the inclusion of AD. However, the [^18^ F] florbetapir PET scan was negative for amyloid, thus preventing a misdiagnosis and potential enrollment in an inappropriate clinical trial of an experimental amyloid-reducing agent (Mitsis *et al*, in preparation).

To provide guidance to dementia care practitioners, as well as patients and caregivers, the Alzheimer’s Association and the Society of Nuclear Medicine and Molecular Imaging convened the Amyloid Imaging Taskforce (AIT) to develop criteria for appropriate use of brain amyloid imaging based upon consensus of expert opinion [11,14,15]. Given the dearth of information on the clinical use and utility of amyloid PET imaging, the criteria offer definitions of the types of patients and clinical circumstances in which amyloid PET imaging can be used reliably as well as circumstances under which the amyloid scans may be unreliable. According to the AIT, uncertainties due to the complexities of patient history and the inconsistencies in examination results could be clarified by the incorporation of amyloid imaging into clinical decision making in order to either clarify the choices amongst various entities in the differential diagnosis and/or to simplify the complexities associated with evaluation [15]. Of course, as with any type of diagnosis, this also requires a careful history, examination, and all other tools necessary and available toward patient care. The AIT warns that amyloid imaging is not equivalent to clinical diagnosis of AD and should be used only as an additional tool.

Amyloid imaging could be used to diagnose patients who present with cognitive complaints, in whom many clinicians reflexively (and nihilistically) assume that the diagnosis is AD. A negative result on [^18^ F] florbetapir PET imaging adds another level of *current* certainty to diagnosis and offers reassurance to the patient and may provoke a more aggressive pursuit of other, potentially reversible causes. In addition, amyloid imaging may serve as a baseline, as positive amyloid scans may be observed in asymptomatic people who may or may not progress to AD. Amyloid deposition precedes cognitive symptoms in familial AD [16] and is believed to do so in most typical sporadic AD [12]. To this end, positive amyloid imaging may be a preclinical marker of the disease [17-19], particularly in normal aging [19]. However, we currently lack the evidence upon which we could reliably and accurately advise such a patient as to whether or not he or she is destined to develop AD, and, if so, when symptoms would be predicted to present themselves. Therefore, AIT advises against amyloid scanning in asymptomatic subjects.

According to the recent United States FDA regulatory guidelines for [^18^ F] florbetapir imaging, a negative scan is useful in excluding an AD diagnosis, while a positive scan is not necessarily definitive. We would agree but argue also that in clinical practice and particularly in the cohort of patients presented herein, a full dementia diagnostic evaluation that considers a variety of biomarkers could be substantially strengthened by the addition of [^18^ F] florbetapir imaging in order to enhance diagnostic certainty and to guide intervention and treatment planning. In some cases, [^18^ F] florbetapir imaging clarified diagnosis in patients presenting with a cognitive profile similar to AD yet had a negative scan (Additional file 1: Table S1, patients 13, 16, 20, 26). In the absence of [^18^ F] florbetapir imaging data, these patients would have been referred to a clinical trial with an anti-amyloid agent, which would have been inappropriate both in terms of prospects of clinical benefit and in terms of reliable evaluation of efficacy of the drugs under evaluation. The recent recognition that sometimes clinical symptoms and neurodegeneration precede imaging evidence for cerebral amyloidosis underscores the importance of amyloid imaging in determining entry into all trials of amyloid-reducing agents [7]. In order to avoid the enrollment of subjects without amyloidosis into trials of amyloid-reducing agents, the Center for Medicare and Medicaid Services (CMS) has agreed to cover one amyloid imaging scan per patient as a part of the trial screening process ( http://www.cms.gov/medicare-coverage-database/details/nca-decision-memo.aspx?NCAId=265). In contrast, the value of [^18^ F] florbetapir imaging in other circumstances has been questioned by the CMS, prompting the recent decision by CMS not to cover routine [^18^ F] florbetapir imaging in Medicare patients ( http://www.cms.gov/medicare-coverage-database/details/nca-decisionmemo.aspx?NCAId=265). The CMS ruling was based on the fact that there is no disease-modifying drug available for the treatment of AD, and that there is currently no evidence that early diagnosis modifies the outcome.

## Conclusions

Unlike research studies based in Alzheimer’s Disease Research Centers and memory clinics that are enriched for patients with likely or probable AD, our experience reflects results from a clinical sample of patients who presented for a comprehensive dementia evaluation at an urban medical practice and were referred for [^18^ F] florbetapir PET imaging for diagnostic identification. It has been our experience with these cases that [^18^ F] florbetapir PET imaging added meaningful diagnostic clarification and certainty. For some patients, that clarification led to a change in their treatment protocol. In some instances, we were able to assist amyloid-positive patients and their families in planning for the future in terms of appropriate care and treatment, as well as long-term personal decision making by the patients themselves. Finally, for individuals who were amyloid positive, we were able to offer information to patients and their families or caregivers regarding the opportunity to enroll in clinical trials of amyloid-reducing agents and other therapeutic agents.

## Methods

### Patient demographics and evaluation

A consecutive case series of 30 patients (age 50-89; 16 M/14 F) who were evaluated clinically and referred for [^18^ F] florbetapir PET imaging for diagnostic confirmation and/or clarification is presented. Patient education ranged between 12 and 20 years (high school graduate to advanced degrees). Patients had a number of comorbid medical conditions and were taking varied medications at the time of [^18^ F] florbetapir PET imaging (Additional file 1: Table S1). All patients underwent comprehensive history acquisition and physical examination by a board-certified neurologist or neuropsychiatrist (SG, MG, or AA). Clinical neuropsychological assessment by PhD level neuropsychologists (HB, JM, JW, and MCS) was conducted on most patients; n = 19). All practitioners were experts in the evaluation and diagnosis of neurodegenerative disorders. Clinical diagnoses were made prior to scanning based upon physician global clinical impression and neurocognitive metrics. One patient had lumbar puncture for extraction of CSF for tau and Aβ42 biomarker analysis. Trained nuclear medicine physicians (LK, JM), using the qualitative, binary visual approach, rated the scans as positive or negative for amyloid. Fifteen patients had had at least one brain MRI within 6 months of the [^18^ F] florbetapir PET imaging (see Additional file 1: Table S1).

### [^18^ F] Florbetapir PET image acquisition

The camera used for [^18^ F] florbetapir PET/CT Brain Imaging was a GE Discovery STE 16-slice PET/CT Camera. Patients were injected with 370 MBq (10 mCi) of [^18^ F] florbetapir. Image acquisition began approximately 60 minutes post injection for all patients. Florbetapir was well tolerated, and there were no adverse events. Images were acquired in 3-D, for 10 minutes, using a one frame and one bed position. Reconstruction was performed with a 120 × 120 matrix utilizing Iterative Reconstruction, with 35 Subsets and 2 Iterations. The Z-axis filter is standard, and the post filter is a 2.57 mm FWHM (*full width*/*half max*) filter. The field of view is set at a 30 cm diameter, with 47 total slices.

### [^18^ F] Florbetapir PET image interpretation

We adhered to the practices in clinical nuclear medicine of binary, visual reading of Amyvid scans and as outlined by the FDA regarding [^18^ F] florbetapir PET imaging (Amyvid®) for these clinical cases. This approach is consistent with the recommendation put forth by both the FDA and Avid Pharmaceuticals (Eli Lilly subsidiary; makers of the ligand) for interpreting florbetapir scans in clinical practice. In fact, to our knowledge it is the only training method for reading of [^18^ F] florbetapir PET imaging (Amyvid®) scans offered by Avid Pharmaceuticals to nuclear medicine physicians. As such, we sought to validate this approach in a typical, heterogeneous sample presenting at our dementia center, as it will be the method used in most clinical settings outside of research. The limitations to using the visual read approach only is that the findings are: 1) in the eye of the rater and 2) we run the risk of not identifying regionally specific findings of amyloid accumulation. It is important to note that our raters were nuclear medicine physicians who underwent intensive training in the visual read approach by the principals at Avid Pharmaceuticals prior to reading the scans. In addition, our raters began by using a consensus approach for the first several patients (n < 5) and were in complete agreement on all the initial cases. Because of the high level of agreement between the two readers, the scan results for the remaining patients are based upon the visual, binary read of each reader independently.

Transaxial, coronal and sagittal images were examined. Uptake in the cerebral cortex was compared to that in the cerebellar and cerebral white matter tracts, both of which normally have high non-specific uptake, while cerebral gray matter normally has low uptake. A study was considered positive if uptake in the cerebral gray matter equaled or exceeded the uptake in the white matter in at least two major areas of the brain. A positive [^18^ F] florbetapir scan indicates moderate to frequent fibrillar amyloid plaques. A negative [^18^ F] florbetapir scan indicates sparse to no fibrillar amyloid plaques and was inconsistent with a diagnosis of AD. A negative scan suggests that a patient’s cognitive decline was not due to AD. Notable potential confounds include: (1) the recent recognition of “neurodegeneration-first” AD [7], the neuropathology of which remains to be established; and (2) the unreliability of amyloid imaging agents to recognize accumulation of oligomeric assemblies of the amyloid-beta peptide [20].

## Competing interests

The authors declare that they have no competing interests.

## Authors’ contributions

EMM contributed to acquisition of data, drafted the manuscript and agrees to be accountable for all aspects of the work in ensuring that questions related to the accuracy or integrity of any part of the work are appropriately investigated and resolved. HB conducted neuropsychological evaluation of patients and provided revisions to initial drafts of the manuscript. JM, MCS, JW conducted neuropsychological evaluation of patients. LK and JM read the results of each patient’s [^18^ F] florbetapir scan and provided feedback to the manuscript. MG and AA provided patients for the series and feedback on manuscript draft. MS participated in the case series design and provided critical feedback to initial drafts. SG participated in the case series design and coordination and provided critical feedback to initial and subsequent drafts. All authors read and approved the final manuscript.

## Supplementary Material

Additional file 1: Table S1 Patient demographics, scan outcomes. Click here for file

## References

[B1] Solomon PR Brush M Identifying dementia in the primary care practice Int Psychogeriatr 2000 12 4 483 493 10.1017/S1041610200006608 11263715

[B2] Lopponen M Raiha I Diagnosing cognitive impairment and dementia in primary health care – a more active approach is needed Age Ageing 2003 32 6 606 612 10.1093/ageing/afg097 14600001

[B3] Wong DF Rosenberg PB In vivo imaging of amyloid deposition in Alzheimer disease using the radioligand 18F-AV-45 (florbetapir [corrected] F 18) J Nucl Med 2010 51 6 913 920 10.2967/jnumed.109.069088 20501908 PMC3101877

[B4] Clark CM Schneider JA Use of florbetapir-PET for imaging beta-amyloid pathology JAMA 2011 305 3 275 283 10.1001/jama.2010.2008 21245183 PMC7041965

[B5] Lister-James J Pontecorvo MJ Florbetapir f-18: a histopathologically validated Beta-amyloid positron emission tomography imaging agent Semin Nucl Med 2011 41 4 300 304 10.1053/j.semnuclmed.2011.03.001 21624563

[B6] Clark CM Pontecorvo MJ Cerebral PET with florbetapir compared with neuropathology at autopsy for detection of neuritic amyloid-beta plaques: a prospective cohort study Lancet Neurol 2012 11 8 669 678 10.1016/S1474-4422(12)70142-4 22749065

[B7] Jack CR Jr Wiste HJ Amyloid-first and neurodegeneration-first profiles characterize incident amyloid PET positivity Neurology 2013 81 20 1732 1740 10.1212/01.wnl.0000435556.21319.e4 24132377 PMC3821718

[B8] Doraiswamy PM Sperling RA Amyloid-beta assessed by florbetapir F 18 PET and 18-month cognitive decline: a multicenter study Neurology 2012 79 16 1636 1644 10.1212/WNL.0b013e3182661f74 22786606 PMC3468774

[B9] Fleisher AS Chen K Apolipoprotein E epsilon4 and age effects on florbetapir positron emission tomography in healthy aging and Alzheimer disease Neurobiol of aging 2013 34 1 1 12 10.1016/j.neurobiolaging.2012.04.017 22633529

[B10] Fleisher AS Chen K Using positron emission tomography and florbetapir F18 to image cortical amyloid in patients with mild cognitive impairment or dementia due to Alzheimer disease Archives of Neurol 2011 68 11 1404 1411 10.1001/archneurol.2011.150 21747008

[B11] Johnson KA Minoshima S Appropriate use criteria for amyloid PET: a report of the amyloid imaging task force, the Society of Nuclear Medicine and Molecular Imaging, and the Alzheimer's Association Alzheimers Dement 2013 9 1 1 16 10.1016/j.jalz.2012.11.006 23360977 PMC3733252

[B12] Sperling RA Johnson KA Amyloid deposition detected with florbetapir F 18 ((18)F-AV-45) is related to lower episodic memory performance in clinically normal older individuals Neurobiol Aging 2013 34 3 822 831 10.1016/j.neurobiolaging.2012.06.014 22878163 PMC3518678

[B13] Albert MS DeKosky ST The diagnosis of mild cognitive impairment due to Alzheimer's disease: recommendations from the National Institute on Aging-Alzheimer's Association workgroups on diagnostic guidelines for Alzheimer's disease Alzheimers Dement 2011 7 3 270 279 10.1016/j.jalz.2011.03.008 21514249 PMC3312027

[B14] Johnson KA Minoshima S Update on appropriate use criteria for amyloid PET imaging: dementia experts, mild cognitive impairment, and education J Nucl Med 2013 54 7 1011 1013 10.2967/jnumed.113.127068 23753186

[B15] Johnson KA Sperling RA Florbetapir (F18-AV-45) PET to assess amyloid burden in Alzheimer's disease dementia, mild cognitive impairment, and normal aging Alzheimers Dement 2013 9 5 Suppl 572 583 23375563 10.1016/j.jalz.2012.10.007 PMC3800236

[B16] Bateman RJ Xiong C Dominantly Inherited Alzheimer Network. Clinical and biomarker changes in dominantly inherited Alzheimer's disease N Engl J Med 2012 30 9 795 804 367 22784036 10.1056/NEJMoa1202753 PMC3474597

[B17] Mintun MA Larossa GN [11C]PIB in a nondemented population: potential antecedent marker of Alzheimer disease Neurology 2006 67 3 446 452 10.1212/01.wnl.0000228230.26044.a4 16894106

[B18] Pike KE Savage G Beta-amyloid imaging and memory in non-demented individuals: evidence for preclinical Alzheimer's disease Brain 2007 130 Pt 11 2837 2844 17928318 10.1093/brain/awm238

[B19] Rosenberg PB Wong DF Cognition and amyloid load in Alzheimer disease imaged with florbetapir F 18(AV-45) positron emission tomography Am J Geriatr Psychiatry 2013 21 3 272 278 10.1016/j.jagp.2012.11.016 23395194 PMC3711793

[B20] Scholl M Wall A Low PiB PET retention in presence of pathologic CSF biomarkers in Arctic APP mutation carriers Neurology 2012 79 3 229 236 10.1212/WNL.0b013e31825fdf18 22700814

