# Supplementary material for: A consecutive case series experience with [18 F] florbetapir PET imaging in an urban dementia center: impact on quality of life, decision making, and disposition
Source: Mol Neurodegener. 2014 Feb 3;9:10. doi: 10.1186/1750-1326-9-10 (PMC3913628; doi:10.1186/1750-1326-9-10)
Supplement: Additional file 1: Table S1 — Patient demographics, scan outcomes. [file 1750-1326-9-10-S1.pdf]

Table 1. Patient demographics, scan outcomes

Patient	Age	Sex	Education	Diagnosis Pre-Scan	Diagnosis Post-Scan	Lessons from Each	[18F]Amyvid (positive vs. negative)	Diagnostic Outcome	MRI	Neuropsychological findings	Medications	Other diagnoses	Amyvid vs. MRI_Days Apart
1	70	F	12	diagnostic dilemma pre Amyvid; given alternate diagnoses by multiple doctors	Clinical syndrome of FTD, behavioral variant due to Alzheimer's pathology	supports the notion that FTD like clinical phenotypes in 70 or over is more likely due to AD	positive	deficits in the areas of verbal fluency, executive functioning, and visuospatial functioning. Memory functioning remains intact; attention and concentration intact	Moderate periventricular T2 abnormality consistent with ischaemic change more marked in the frontal lobes. Mild to moderate generalized cerebral atrophy but no other focal lesions. Dominant right vertebral artery but MRA otherwise satisfactory	history of cognitive and behavioral changes; Diagnosed elsewhere with AD; Diagnosis unclear due to long-term alcohol abuse (R/O alcohol dementia), Diagnosis probable Dementia, Not Otherwise Specified, With Behavioral Disturbance	Temazepam; Levothyroxine; Pantoprazole; Sertraline; Donepezil (Aricept), 5mg	Hypothyroidism (not consistently treated due to medication non-compliance), and repeated urinary infections.	MRI was performed 185 days before Amyvid
2	80	M	18	probably MCI due to AD	static memory disorder	unexpected negative; amnestic syndrome static for 2+ yrs; neuropsychological testing, with knowledge Amyvid was negative, suggested vascular but vascular signature not clear. Major revision in pre-Amyvid diagnosis of probable MCI due to AD	negative	static memory impairment, follow up in 1-2 years; cognitive remediation recommended	mild, diffuse atrophy, with parietal and mesiotemporal predominance	Attentional dysfunction, primarily due to slowed speed of information-processing, almost certainly contributing to inefficient learning and patient's subjective complaint of memory dysfunction. Presentation consistent with effects of both normal aging and small vessel ischemic changes. No diagnosis provided; cognitive remediation and 1-2 year f/u recommended	Digoxin, Donepezil, Lipitor, Metoprolol, Warfarin Avodart, Nexium, Uroxatral, Vitamin D3, MVI, Ferrous sulfate, Saw palmetto, Osteo-biflex	Aortic aneurysm, AFIB, CAD, History of DVT, Hyperlipidemia, BPH, Lymphoma 2003, no apparent residual disease, GERD, Keratocystic odontogenic tumor, Benign essential tremor	MRI was performed 9 days after Amyvid
3	60	F	12	AD vs FTD	FTLD-PPA/vascular	typical PPA FTD	negative	diagnostic clarification	Focal brain volume loss involving the left frontal lobe associated with reduced metabolic activity on the FDG PET scan. Verifies chronic ischemic change. Central and left-sided ex vacuo volume loss. Minimal increased signal within the centrum semiovale, corona radiata, and periventricular white matter compatible with senescent change. No evidence of recent infarction or hemorrhage. No evidence	Gradual decline in cognitive abilities; Specific difficulties included acaculia, diminished verbal fluency, decreased attention and concentration, and confrontation naming deficits. Subjective sense of severe deficits in the areas of learning and memory; loses important items and forgets conversations, dates, and events. Was diagnosed with frontotemporal degeneration elsewhere. Current findings: decrements in confrontation naming, aspects of executive functioning, and verbally and visually presented learning and memory. Executive dysfunction was characterized by diminished abstraction, organization and planning difficulties, set-shifting deficits, reduced verbal fluency, and diminished psychomotor speed. Rapid rates of forgetting following a delay.	Galantamine 8mg, Extended release pellets 24 hr Wellbutrin XL300mg 24 hr tablet; Namenda 10mg; Lexapro 10mg; esCITalopram 5mg; sulfamethoxazole-trimethoprim 800-160mg; Bupropion XL150mg 24 hr tablet; nabumetone 500mg		MRI was performed 154 days after Amyvid
4	67	F	18	FTD	FTD-PPA	FTD-PPA	negative		Impression: Focal volume loss left fromtal lobe associated with reduced metabolic activity. No evidence of recent infarction, hemorrhage, or hydrocephalus.	Severe deficits in memory and learning abilities, language, processing speed and executive functioning. Significant weaknesses in executive functioning, including, set-shifting, planning, and organizing abilities Relatively well-preserved abilities in working memory and visuospatial processing. mild levels of depression and anxiety. NP findings indicate the presence of a cortically-based primary neurodegenerative disorder. Clinical history and significant aphasia is consistent with a diagnosis of primary progressive aphasia; however, the patient's diffuse cognitive deficits appear to be greater than what would be expected in PPA alone.	Ambien PRN		MRI date is not available on EPIC/NP report
5	81	M	16	PD	AD with CAA	diagnosed as PD because of bradykinesia rigidity, then had stroke, was most likely AD with extrapyramidal then CAA bleed, major revision in pre-Amyvid diagnosis	positive	major revision to pre-scan diagnosis	Ischemic changes that are slightly more prominent than a previous study. No focal interval abnormality.	Cognitive testing not done; patient babbles incoherently.	Sulfamethoxazole-trimethoprim 400-80mg; Ciprodex 0.3-0.1% Drops, Aricept 10mg; Synthroid 75mcg; desonide 0.05 % Top Ointment; ketoconazole 2 % topical cream; nystatin 100,000 unit/g; Iron Polysacch Complex-B12-FA 150-25-1mg-mcg-mg; ropinirole 0.5mg; carbidopa-levodopa 50-200 mg; Felodipine 2.5 mg Oral Tb24; Ambien 5mg; Alfuzosin 10mg; Prilosec 20mg; Tramadol 50mg; Colace100mg; Calcium Carbonate 500 mg (1,250mg);		MRI was performed 455 days before Amyvid
6	79	F	16	Subjective cognitive complaint	worried well	e4 worried well; negative scan interpreted as favorable portent; i.e., safe for 10 years more?	negative		No MRI	No neuropsychological testing conducted.	Temazepam; Levothyroxine; Pantoprazole; Sertraline; Aricept		no MRI
7	78	M	12	AD	AD		positive		Previous imaging was unremarkable.	Gradually worsening cognitive functioning over the past year. Specific complaints include forgetfulness and mild confusion; Needs reminding several times to complete a task. No changes in graphomotor skills or vision were reported. Motor skills are intact. The patient further denies any perceptible change in processing speed or ability. Accuracy with finances has diminished. Brief neuropsychological screen showed widespread dysfunction and fell below the cutoff for dementia. Learning and memory deficient; Executive dysfunction; fine motor coordination below expectation, bilaterally	Avapro 150 mg, Lipitor 20 mg, Neurontin 30 mg, Alpha Lipoic 100 mg		MRI was performed 42 days before Amvyid
8	75	F		AD	PD+Depression	Amyvid excluded AD but AD not very likely from start	negative		No MRI	No neuropsychological report available.	No medications		no MRI
9	74	M	12	FTD, PPA	FTLD, Behavioral variant	typical PPA FTD	negative		Mild white matter changes in the frontal regions. No evidence of acute infarct, mass effect or intracranial hemorrhage. No abnormal enhancement is seen	Tested in a foreign language. Results not available.	No medications		MRI was performed 30 days after Amvyid

10	89	F	12	AD	AD		positive		No MRI	premorbid intellectual abilities at least within the high average range. Scores on an abbreviated measure of intellectual functioning indicated that current level of global intellectual abilities was below this estimate; verbal skills were a relative strength compared to nonverbal capacity. On tests of overall cognitive functioning, performance was low average to average. Well preserved abilities in the areas of language, attention, processing speed, and executive functioning. Neuropsychological weaknesses on tasks of verbal and nonverbal memory; a relative weakness on visuospatial abilities	multiple vitamin supplements, including: glucosamine, vitamin C, a multivitamin, citrate with vitamin D, fish oil, vitamin E, vitamin B-12, biotin, cholecaleiferol, among others. One-half a Tylenol PM for sleep. Was prescribed Aricept but recently stopped taking due to GI side effects.		no MRI
11	83	M	15	AD	AD	typical AD pre and post	positive		age-related atrophy with minimal periventricular white matter change. The temporal horns and anterior temporal tips show no inordinate atrophy	Estimated level of pre-morbid intellectual functioning High Average to Superior range. Patient oriented to person, place, and time. Results of neuropsychological testing indicate a modest decline in memory functioning for both verbal and visual information. Unable to encode and retrieve newly learned information at a level consistent with pre-morbid intellectual ability. Language functioning and verbal fluency within normal limits for age. Attention and executive functioning were variable and contributed to weak performance in other cognitive domains. Visuospatial functioning was intact	Micardis, Crestor, Nexium, Atenolol; Xanax PRN	hypertension, medically controlled. Recent increased anxiety.	MRI was performed the same day as Amyvid
12	77	M		AD	AD	typical AD pre and post	positive		Old lacunar infarctions. Multiple punctate foci of chronic hemorrhage distributed within the cerebral hemispheres compatible with a hemorrhagic angiopathy such as hypertension or amyloid. No evidence of recent [illegible]	Premorbid level of abilities estimated to be within the High Average range. The testing results obtained largely fell well below this estimate, demonstrating widespread neuropsychological impairment. Overall, diminished memory performance, poor attention, decreased initiation, and reduced information processing.	Donepezil 10mg; Diovan 80mg; Lipitor 10mg; Aspirin 81mg		MRI was performed 6 days before Amyvid
13	77	F	18	AD? To unknown?	depression/MCI	suspected AD but negative Amyvid; normal FTD probably Bipolar I or pseudodementia	negative	negative but presented with AD profile	No evidence of lobar infarct, hemorrhage, mass or hydrocephalus. Moderate chronic small vessel ischemic change	Repeating self in conversation, which was a marked change from baseline level of abilities. Frequent falls 6-12 months ago. The patient denied coordination difficulties. Denied any loss of consciousness or concussions. Neurological and cardiology workups were inconclusive regarding the etiology of falls. Psychiatric history is significant for severe depression and psychiatric hospitalization in previous year. Was treated with Klonopin. Estimated premorbid abilities are within the High Average range. Current level of functioning is Average, indicating a decline from premorbid levels; Significantly stronger verbal than non-verbal abilities. Significant and generalized memory impairment, decreased attention, executive dysfunction, and reduced fine motor speed. Impaired encoding and delayed recall abilities for unstructured verbal information. Executive dysfunction. Language abilities, visuospatial abilities, and simple attention were all intact and within normal limits. Fine motor coordination was impaired bilaterally. Patient denied significant depressive and anxiety symptomatology during the clinical Interview, and on structured questionnaires, yet affect during the evaluation suggested the presence of moderate depression and anxiety.	Aspirin, Centrum, Claritin, Colace, Diovan, Effexor, Klonopin, Melatonin, Norvasc, Omeprazole, Pravachol, Mirtazapine, and Senna		MRI was performed 126 days before Amyvid
14	71	F	15	AD	AD	typical AD pre and post	positive		Significant for small vessel disease	Significant deficits were found in memory and new learning for both visual and verbal information. An executive component to the memory deficits, as patient has difficulty organizing both verbal and visual information for effective retrieval. Performance was variably poor on executive measures that required cognitive flexibility and mental set shifting. Memory and executive deficits not attributed to psychological distress and are likely organic in nature. On CVLT, recalled no words after 20 min delay. Progressive process may be difficult to distinguish between small vessel disease vs. AD.	MVA; prescribed Aricept 5mg but had not taken it at time of NP and Amyvid.		MRI performed 518 days before Amyvid
15	72	M	18	AD	AD	typical AD pre and post	positive		No MRI	General intellectual function High Average to Superior; impaired memory; Recall defective; has degree of depressive symptomotology however pattern and extent of cognitive dysfunction not likely caused by emotional factors.	No medications		no MRI
16	71	M	16	AD (experts disagreed)	DPTCI due to possible CTE	clinical AD changed to probable CTE post Amyvid [illegible]	negative	negative but presented with AD profile; experts disagreed upon including AD	Volume loss globally and in particuar involving the hipposcampal formations on either side. Arachnoid cysts in the middle fossa bilaterally, nonspecific in etiology. Mamillary bodies are poorly seen and may be atrophic but hypothalamic/infundibular/pituitary axis appears preserved. No evidence of acoustic neuroma.	Experienced numerous concussions. Unable to estimate how many times. Does not recall LOC but was dazed and confused for up to a full day following these injuries. Currently, memory is significantly worse, more so in the past year; more easily agitated. Got lost driving in an unfamiliar place; increased agitation. Current findings: poor purdue and grooved pegboard performance with asymmetric findings dominant and non-dominant hand; CPT normal; low average FAS but 6th percentile animal naming; BNT deficient at 1st percentile. Neuropsychological profile consistent with history of TBI and does not suggest AD. Experts disagreed and advocated an AD process, in addition to TBI. Amyvid clarification.	Aricept and Namenda past one year; moderate drinking.		MRI performed 79 days after Amyvid

17	77	M	18	AD	AD	typical AD pre and post Amyvid	positive	was MCI -converted	MRI of the brain w/o contrast done in 2008, approximately 3 years prior to Amyvid scan. There was no evidence of acute infarction. Dilated perivascular space of small cyst (measuring 7mm in length) in the right parietal white matter, otherwise unremarkable examination. Ventricles are normal in size and midline. Overlying cortical sulci are normal. no evidence of hemorrhage, mass-effect or extra-axial collection.	Primary memory disorder with memory functioning far below expectations based upon premorbid level of Superior intellectual functioning. Memory testing was consistent with prior testing in 2010 and did not indicate further decline.	Adderal, Aricept, 81mg Aspirin, Tylenol PM	FDG PET imaging in 2011: Images show globally decreased FDG uptake in cerebral cortex with relative sparing of the occipital lobe and sensorimotor cortex. FDG uptake spared in the basal ganglia. There is a focal area of increased FDG uptake in the right frontoparietal region near the righ sensorimotor cortex, probably artifactual in nature. Impression: Findings may represent AD, although not demonstrating a typical patter of AD. However, globally decreased FDG metabolism with sparing of the basal ganglia and sensorimotor cortex may be seen in AD.	MRI was performed 1480 days before Amyvid
18	65	F		AD	AD	typical AD pre and post	positive		No MRI	NP report is not available	No medications		no MRI
19	50	M	18	Limbic Encephalitis	AD	LE pre-Amvid; AD post Amyvid scan	positive (PiB)		8//2012: IMPRESSION: Senescent vs. small vessel related deep white matter ischemic change. No evidence of recent infarction, hemorrhage or hydrocephalus. 11/2012 Impression: Unremarkable.	Current results suggest qualitative decline in attention, reading, vocabulary, language fluency, and naming. Cuing was not beneficial on any memory test as it had been in the past. Current MMSE was 10/30. All test score in the impaired range. Maintains excellent social skills and sense of humor despite considerable difficulty with verbal expression. Long-term memory preserved.	No medications		MRI was performed 7 days after Amyvid
20	59	M	20	FTD	FTD, behavioral variant; [18F] florbetapir positive only focally at site of impact	post TBI rapidly progressive AD pre Amyvid; FTD with focal Amyvid post Amyvid scan	negative; Amyvid positive only focally at impact	presented with FTD+AD profile	MRI of the brain revealed recent and chronic bleeding over the left hemisphere convexity.	Sports activity related head injury previous year. Persistent headache; no LOC. Subdural hematoma. An MRI of the brain revealed recent and chronic bleeding over the left hemisphere convexity. Had craniotomy to evacuate the subdural hematoma. Experiences hand numbness and tingling. Following craniotomy had focal seizures, which was treated with Keppra but had been weaned off of several months prior to testing. Has some urinary frequency and difficulty with sleep. Neuropsychological assessment was sought to compare results with a previous evaluation performed at another institution prior to Amyvid scan at Mount Sinai due to cognitive changes post brain surgery within the same year. Was previously diagnosed with FTD. Results of neuropsychological testing indicated significant deficits in memory, language, and executive functioning, with performance on most cognitive measures far below expected levels (ranging from Significantly Impaired to High Average). Results were consistent with previous test results suggestive of FTD.	Lipitor, 20mg		MRI date is not available on EPIC/NP report
21	53	M			worried well	e4 worried well; negative scan interpreted as favorable portent; i.e., safe for 10 years more?	negative		No MRI	No neuropsychological evlaluation available.	No medications		no MRI
22	82	F	13	vascular dementia	vascular dementia	VaD pre and post	negative		small vessel related deep white matter ischemic change with old lacuar infarctions. Ventricular dilatation - while this may represent volume loss, the possibility of an extraventricular hydrocephalus cannot be excluded. No evidence of recent infarction or hemorrhage.	Premorbid intellectual functioning is estimated to have been at least in the average range. Limited testing was performed because the patient was resistant to put forth adequate effort in the testing. However, significant cognitive deficits are evident across all domains even given the limited testing performed. The patient scored a 10/30 (0/3 delayed recall) on the MMSE. Immediate and delayed recall of stories was impaired. She was unable to perform Trails A, could not draw a clock, and unable to draw two interlocking pentagons on the MMSE. Animal naming was impaired. Similarities was in the borderline range. A possible right visual field cut was evident on the paper & pencil visually-mediated tasks, which may be consistent with a vascular component contributing to her dementia. Significant functional decline is reported (e.g. required assistnace for all of ADL). Dx of best fit is probable Vascular Dementia.	Metformin; Actos; Prandin; Exelon; Metoprolol succinate; Vitamin D, C; multivitamins; 81mg asprin; Lipitor, 40mg; Plavix, 75mg; levothyroxine Sodium, 88mg		MRI was performed 7 days before Amyvid
23	63	F	18	AD	AD	typical AD pre and post	positive		No evidence of acute infarct, hemorrhage or hydrocephalus.	Premorbid intellectual functioning is in the average range. There is a significant discrepancy between verbal skills and non-verbal skills, with much stronger verbal skills than non-verbal reasoning skills. While attention and concentration (working memory) is in the average range, processing speed of information, particularly visual information, is in the borderline range. NP testing revealed evidence of a memory deficit. The patient was able to encode contextual verbal information (stories) at expected levels (average), but performed in the impaired range in a more challenging memory task of non-contextual information. Visual memory was impaired. Verbal fluency is intact but confrontation naming is impaired. Significant deficits in visual spatial functioining and executive functioning are evident from testing. There is reported functional decline (e.g. word-finding difficulty, forgetting conversations, misplacing things, being significantly less organized, forgets to perform tasks at work). The pattern of test results, clinical interview, medical consultation, reported work complaints, and history of a progressive cognitive decline are consistent with a diagnosis of a probable dementia of the alzheimer's type, without behavioral disturbance, with early onset.	Wellbutrin		MRI was performed 20 days before Amyvid
24	61	M			MCI due to substance abuse		negative	presented with AD profile	No evidence of lobar infarct, hemorrhage or mass.	N/A	No medications		MRI was performed 15 days before Amyvid

25	53	M			MCI		positive	Ventricular prominence. Multifocal susceptibility which might be compatible with either shearing injury, embolic disease, or both. No acute changes, recent infarction, or recent hemorrhage.	N/A	Clonazepam 0.5 mg; lisdexamfetamine 30 mg capsule; galantamine 24 mg capsule,ext rel. pellets 24 hr; bupropion XL 300 mg Oral 24 hr tablet; Memantine 5-10 mg Oral tablets,dose pack; donepezil 5 mg Oral tablet; aspirin 325 mg Oral Tab; metoprolol tartrate 25 mg Oral	MRI was performed the same day as Amyvid
28	53	F			?		negative	T2 weighted images shows biparietal loss of sulcal volume analogous to that seen on CT but more obvious on the MR with a greater involvement in the left parietal region as compared to the right. The calvarium also appears thicker on the left than on the right. On the axial T2s, again these findings are apparent, again with the left side being greater than that on the right. There is a diminishment in the size and number of sulci in the region with loss of volume and greater thickness of the calvarium again noted on the left. This would suggest a longstanding process with remodeling of the bone. Conceivable that this is congenital or acquired early in patient's development	N/A	Pentoxifylline 400 mg tablet extended release level; Tiracetam 500 mg Oral tablet; gabapentin 100 mg Oral Cap; metoprolol succinate XL 50 mg Oral Tb24; atorvastatin 20 mg Oral Tab; carisoprodol 350 mg Oral; buspirone 7.5 mg Oral Tab; lidocaine 5 %(700 mg/patch)	MRI was performed 345 days before Amyvid
26	81	F		AD	AD	typical AD pre and post	positive	No MRI	N/A	Razadyne ER 8mg; extended release pellets 24h	no MRI
27	71	F	12	AD	AD	typical AD pre and post	positive	No MRI	Pre-morbid intellectual functioning is in the average range. NP testing indicate a significant decline in memory and language functioning. Attention was variable and affected by anxiety. Visuospatial functioning was variable and affected by executive functioning difficulties. Executive functioning was variable with some decline evident. There is significant functional decline (e.g. difficulty remembering to pay bill payments, attend appointments, and difficulty operating computer). Test results and clinical history are consistent with dementia. Dx of best fit is probable dementia of the alzheimer's type, late onset, without behavioral disturbance.	aricept, Namenda	no MRI
28	62	M		Subjective cognitive complaint	worried well	e4 worried well; negative scan interpreted as favorable portent; i.e., safe for…10 yrs more?	negative	No MRI	No neuropsychological evaluation available	Ambien 10mg	no MRI
29	56	M		Subjective cognitive complaint	worried well	e4 worried well; negative scan interpreted as favorable portent; i.e., safe for…10 yrs more?	negative	No MRI (unremarkable per outside report)	No neuropsychological evaluation available	No medications	no MRI
30	62	F	20	AD	pseudodementia/depression/BP-I	revision of diagnosis	negative	No MRI	Long history of depression and reported cognitive changes over the past few years. NP finding revealed that the patient is performing below expectation in a range of cognitive domains, including visual memory, working memory, attention and concentration, and processing speed. The patient's intellectual functioning also appears to be somewhat weaker than what would be expected given her educational and occupational background. Results indicate a dementia syndrome of depression, or reversible dementia. AD is unlikely	Lipitor, Amitiza, Dyazide, and Reglan	no MRI
